# DRI-Grass: A New Experimental Platform for Addressing Grassland Ecosystem Responses to Future Precipitation Scenarios in South-East Australia

**DOI:** 10.3389/fpls.2016.01373

**Published:** 2016-09-20

**Authors:** Sally A. Power, Kirk L. Barnett, Raul Ochoa-Hueso, Sarah L. Facey, Eleanor V. J. Gibson-Forty, Susan E. Hartley, Uffe N. Nielsen, David T. Tissue, Scott N. Johnson

**Affiliations:** ^1^Hawkesbury Institute for the Environment, Western Sydney University, PenrithNSW, Australia; ^2^Department of Evolution and Ecology, University of TübingenTübingen, Germany; ^3^School of Biosciences, Cardiff UniversityCardiff, UK; ^4^Department of Biology, York Environment and Sustainability Institute, University of YorkYork, UK

**Keywords:** climate extremes, community ecology, drought, NPP, plant-herbivore interactions, rainfall manipulation, root herbivory

## Abstract

Climate models predict shifts in the amount, frequency and seasonality of rainfall. Given close links between grassland productivity and rainfall, such changes are likely to have profound effects on the functioning of grassland ecosystems and modify species interactions. Here, we introduce a unique, new experimental platform – DRI-Grass (**D**rought and **R**oot Herbivore **I**nteractions in a **Grass**land) – that exposes a south-eastern Australian grassland to five rainfall regimes [Ambient (AMB), increased amount (IA, +50%), reduced amount (RA, -50%), reduced frequency (RF, single rainfall event every 21 days, with total amount unchanged) and summer drought (SD, 12–14 weeks without water, December–March)], and contrasting levels of root herbivory. Incorporation of a belowground herbivore (root-feeding scarabs) addition treatment allows novel investigation of ecological responses to the twin stresses of altered rainfall and root herbivory. We quantified effects of permanently installed rain shelters on microclimate by comparison with outside plots, identifying small shelter effects on air temperature (-0.19°C day, +0.26°C night), soil water content (SWC; -8%) and photosynthetically active radiation (PAR; -16%). Shelters were associated with modest increases in net primary productivity (NPP), particularly during the cool season. Rainfall treatments generated substantial differences in SWC, with the exception of IA; the latter is likely due to a combination of higher transpiration rates associated with greater plant biomass in IA and the low water-holding capacity of the well-drained, sandy soil. Growing season NPP was strongly reduced by SD, but did not respond to the other rainfall treatments. Addition of root herbivores did not affect plant biomass and there were no interactions between herbivory and rainfall treatments in the 1st year of study. Root herbivory did, however, induce foliar silicon-based defenses in *Cynodon dactylon* and *Eragrostis curvula*. Rapid recovery of NPP following resumption of watering in SD plots indicates high functional resilience at the site, and may reflect adaptation of the vegetation to historically high variability in rainfall, both within- and between years. DRI-Grass provides a unique platform for understanding how ecological interactions will be affected by changing rainfall regimes and, specifically, how belowground herbivory modifies grassland resistance and resilience to climate extremes.

## Introduction

Grasslands cover more than 40% of the Earth’s land surface (LeCain et al., 2002). They support tremendous biodiversity, underpin grazing and animal production, and store more than one-third of global terrestrial carbon stocks (Trumper et al., 2009). Given the close relationship between rainfall and both the productivity and diversity of grasslands (Sala et al., 1988; Walter et al., 2012), future changes in rainfall regimes are likely to have a substantial impact on the ability of grasslands to provide these important ecosystem services.

Climate models predict changes in the overall amount and seasonality of rainfall, and increased intervals between rain events (i.e., reduced rainfall frequency; Easterling et al., 2000; Fischer et al., 2013; Intergovernmental Panel on Climate Change [IPCC], 2013). Of particular note is the expectation that prolonged and more intense droughts, in combination with warmer temperatures, will combine to expose ecosystems to more frequent extreme climates, pushing today’s ecosystems into uncharted climate territory (Kayler et al., 2015). The seasonality of rainfall inputs is also a crucial determinant of grassland dynamics, with seedling establishment, productivity and senescence all influenced by the amount and timing of growing season rainfall (Huxman et al., 2004). Indeed, even small increases in winter rainfall have been shown to influence the functioning of grassland ecosystems in the following spring (Fry et al., 2014a). Furthermore, there is a growing body of evidence that reductions in the frequency of rainfall events are at least as (and sometimes more) important as reductions in the size of events, in terms of their effects on key ecological processes (Fay et al., 2003; Knapp et al., 2008; Heisler-White et al., 2009; Peng et al., 2013).

Shifts in rainfall regimes are not only expected to have a major impact on the composition and functioning of grasslands (Fry et al., 2016), but are also likely to modify interactions between plants and their associated herbivores (Staley et al., 2007; Johnson et al., 2011; Lee et al., 2014). Invasive root-feeding scarab beetles were accidentally introduced to Australia in the first part of the 20th century (recently reviewed by Frew et al., 2016) and, in pastures, their collective mass can exceed that of mammals grazing aboveground (Britton, 1978). Because root herbivory is hidden and occurs by attrition, losses in primary productivity are less conspicuous than those due to aboveground herbivory, but can be up to 25% in grassland systems (Seastedt and Murray, 2008). Even minor root herbivory can damage plants and alter their physiology by: (i) decreasing nutrient and water uptake, (ii) causing disproportionate resource losses by severing roots, (iii) diverting assimilates away from shoot growth for root re-growth, (iv) imposing leaf water deficits, and (v) causing infection (Johnson and Murray, 2008; Zvereva and Kozlov, 2012). The resulting effects on plant biomass and metabolism are often larger (Meyer et al., 2009) and differ from those caused by aboveground herbivores (Zvereva and Kozlov, 2012). Impairment of root function via root herbivory has parallels with water stress imposed via periods of drought. Indeed, a recent meta-analysis has shown that root herbivory and drought reduced plant growth to a greater extent than any other combination of biotic and abiotic stresses (Zvereva and Kozlov, 2012). Moreover, root herbivory can change plant community composition in grasslands via preferential feeding on certain plants (Schallhart et al., 2012).

DRI-Grass (**D**rought and **R**oot Herbivore **I**nteractions in a **Grass**land Ecosystem) is a new experimental platform designed to examine ecosystem responses to the twin stresses of altered rainfall and root herbivory. Uniquely, DRI-Grass includes shifts in the size, frequency and seasonality of rainfall events, and incorporates a factorial belowground herbivore addition treatment to investigate interactions between these abiotic and biotic stresses. It joins a new generation of drought experiments (*sensu* Thompson et al., 2013) that incorporate realism in terms of both future rainfall scenarios (e.g., Jentsch et al., 2007; Hoover et al., 2014; Knapp et al., 2015) and also trophic complexity (Johnson et al., 2011, 2015; Zhu et al., 2014). Despite the clear importance of root herbivores for the functioning of grassland ecosystems (Frew et al., 2016), their role in moderating grassland resistance and resilience under changing rainfall regimes has rarely been examined in long term field-scale experiments.

Here we introduce DRI-Grass, presenting microclimatic data that demonstrate the impacts of shelter infrastructure on the physical and biotic environment. We also present data on early vegetation responses to test the hypotheses that: (1) reduced rainfall amount and summer-long drought, will reduce aboveground productivity to a greater degree than a shift in rainfall frequency toward fewer, larger events (with annual rainfall amount unchanged); and (2) root herbivory will alter plant quantitative (e.g., ANPP) and qualitative (e.g., chemical) responses to altered rainfall regimes. In focusing on our approach and methodology, this paper aims to provide the methodological detail that will assist other researchers interested in constructing experimental platforms that incorporate both biotic and abiotic stressors. Presentation of selected early results is intended to provide a broad indication of the ecosystem responses that can be measured using this multi-stressor, multi-trophic approach.

## The DRI-Grass Experimental Platform

The study site is located in Richmond, NSW, Australia (S33 36′35, E150 44′18), at an elevation of 25 m a.s.l. Mean annual rainfall at the site is 806 mm (Australian Government Bureau of Meteorology, Richmond – UWS Hawkesbury Station^1^), with summer being the wettest season and winter generally the driest. Seasonal mean maximum/minimum temperatures are 29.4/18.8°C in summer and 17.3/3.2°C in winter. The soil is a Blackendon Sand, with a sandy loam texture and a water holding capacity of 20–22%. There is a mineral hardpan present at approximately 90 cm depth. **Table 1** summarizes the soil characteristics of the site.

**Table 1:** Soil properties at the DRI-Grass field site.

Soil property	Value (units)
Texture	84.4% sand
	7.4% silt
	9.2% clay
SOM content	2.4%
pH	6.4
Total N	0.011 mg g^-1^
Total P	0.0016 mg g^-1^
^∗^Exchangeable NO_3_	17.1 μg cm^-2^ 90 days^-1^
^∗^Exchangeable NH_4_	3.6 μg cm^-2^ 90 days^-1^
^∗^Exchangeable PO_4_	1.55 μg cm^-2^ 90 days^-1^
Bulk density	1.66 g cm^-3^
C:N ratio	12.98
Water holding capacity	0.21 ml ml^-1^

*^∗^Exchangeable nutrient concentrations obtained using ion exchange membranes (Plant Root Simulators)^®^; values measured at 0–10 cm depth.*

The experiment is situated within a former pasture grassland, comprising a total of 62 plant species (Supplementary Table S1), of which ∼12 species are common. The most abundant species include the C_4_ grasses *Axonopus fissifolius, Cynodon dactylon, Cymbopogon refractus, Eragrostis curvula*, and *Paspalum dilatatum*, the C_3_ grasses *Microlaena stipoides* and *Lolium perenne*, and the C_3_ forbs *Hypochaeris radicata* and *Plantago lanceolata*. The site had been under grazing management until 2001; since this time grazers were removed, the site was fenced and subsequently mown every 2–3 months, until the experiment commenced in June 2013.

## Rainout Shelter Design

**FIGURE 1:**
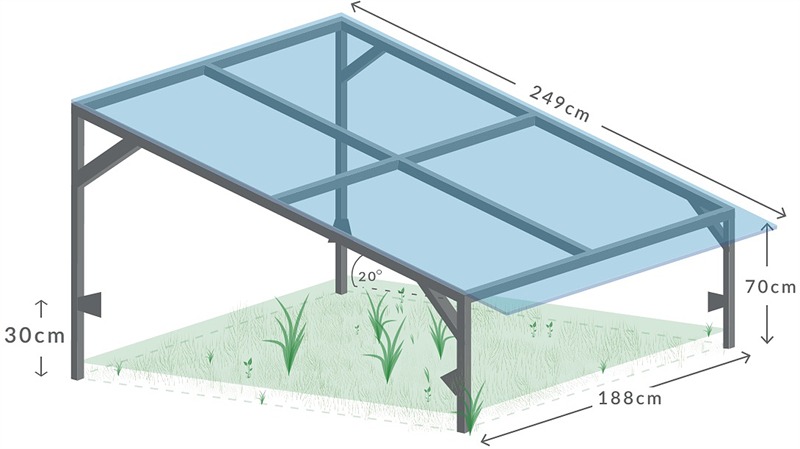
**Schematic of DRI-Grass rainout shelter design**.

Shelter frames are made from 25 mm galvanized steel tubing and covered with a single sheet of clear Acrylic cast Perspex (1.88 m × 2.49 m, Mulford Plastics, Silverwater, NSW, Australia). Roofs are at a maximum height of 140 cm, sloping at a 20° angle down to a low-end height of 70 cm (**Figure 1**). Shelters are orientated along a SW-NE axis, with the low end facing into the direction of the prevailing wind. All rainfall is intercepted and directed away from the plots. Water treatments are applied following each rainfall event, using an irrigation system controlled by a Campbell logger (CR1000) and a series of 16-Channel AC/DC Relay Controller units (SDM-CD16AC units; Campbell Scientific, Thuringowa, QLD, Australia) that control solenoid valve opening/closure, and thus regulate delivery of water to individual plots. To simulate rainfall patterns that reflect actual rainfall events, the amount of water delivered is proportionate to the amount of precipitation that has fallen in the previous 24 h (i.e., AMB receives the same amount of rainfall as measured at the site in the previous 24 h; IA receives 50% more; and RA receives 50% less than the ambient amount). Target amounts of water are set using a calibrated flow meter. Water is delivered to each plot via a network of polyethylene pipes and four 90° spray heads per plot, mounted at a height of 30–45 cm (moveable, depending on vegetation height) at the corners of each shelter. An impermeable root barrier is installed within each plot, just inside the roof footprint, to a depth of 30 cm, giving an actual plot size of 1.8 m × 2.0 m (i.e., 3.6 m^2^). This barrier prevents incursion of roots from outside the experimental plots and minimizes horizontal water flow between plots and the surrounding grassland area.

## Environmental Monitoring

Rainfall is measured using a tipping bucket rain sensor (0.2 mm graduation, ICT International, Armidale, NSW, Australia) and air temperature is measured on site every 5 min (model 107 sensor, with radiation shield, Campbell Scientific, Thuringowa, QLD, Australia). Photosynthetically active radiation (PAR) is recorded at 15 min intervals (Apogee sensors, model SQ-110, ICT International, Armidale, NSW, Australia), under three shelters and in three unsheltered (outside) plots.

Soil moisture TDR probes (CS616, Campbell Scientific, Thuringowa, QLD, Australia) with 30 cm long prongs are installed at an angle of 30°, to integrate moisture readings for the top 15 cm of the soil profile, in half of the plots (*n* = 3 per treatment combination). Regular (approximately every 4–6 weeks) measurements of soil moisture are also conducted manually in all plots, using a theta probe (Delta T Devices, UK), to determine whether automatically logged moisture readings from permanently sensored plots are representative of the respective treatments.

Given the open-sided nature of the shelters and the potential for rain ingress under windy conditions, edge effects on soil moisture were quantified under a range of conditions, including during dry periods and after small, medium and large rainfall events. Soil moisture (0–10 cm depth) was measured using a theta probe inserted in a 5 × 5 grid system, covering 25 points per plot, evenly spaced at a distance of 40 cm from the plot boundary and 40 cm from the next grid point. These within-plot measurements were compared with readings taken immediately outside of the shelters (eight replicates – two along each side of the plot).

## Experimental Design

The experiment comprises five different rainfall treatments, three of which are crossed with a root herbivory treatment (detailed below). All treatment combinations are replicated six times, in a fully randomized block design [*n* = 48 (i.e., 8 × 6) for sheltered plots]. There are also 12 unsheltered plots [hereinafter referred to as “Outside Plots (OP)”] – six with herbivore additions and six without the addition of herbivores – making a total of 60 experimental plots.

Rainfall treatments comprise: (a) sheltered control (AMB), (b) reduced rainfall amount (RA: 50% reduction of ambient), (c) reduced rainfall frequency (RF: ambient rainfall amount, as a single application once every 21 days), (d) increased rainfall amount (IA: 50% increase of ambient), and (e) summer drought (SD: complete removal of all rainfall for a 12–14 weeks period, December–March, with ambient rainfall thereafter). Unsheltered (outside control) plots (OP) receiving ambient rainfall were also included to evaluate the magnitude of shelter effects. Rainfall treatment effects were assessed by comparing the four altered scenarios (RA, RF, IA, and SD) to the sheltered control plots (AMB). Rainfall treatments commenced on June 21, 2013.

Root herbivore treatment: Three of the rainfall treatments (AMB, RA, and RF) and OP also include a belowground herbivore addition treatment (*n* = 6 for each treatment combination). To impose the herbivore addition treatment, 27 g of locally collected adult scarab beetles (Coleoptera: Scarabaeidae) were added to the herbivore addition plots in December 2013, and an additional 9 g of adult beetles were added to each plot in February–March 2014. Adult beetles were added to plots by placing them within mesh enclosures in the plots, and allowing them to oviposit for a period of 3 days on each occasion, before mesh enclosures were removed. In order to control for the effects of the mesh enclosures on vegetation, identical structures were placed on paired (herbivore-free) plots at the same time. We verified the efficacy of herbivore treatments 18 months after beetle additions (October 2015) via destructive, within-plot soil excavation and associated sampling. This involved excavating two holes (25 cm × 10 cm) per plot to a depth of 20 cm; samples were separated into two depths: 0–10 and 10–20 cm, and sieved. Macro and mesofauna were collected, identified under a dissecting microscope and counted.

## Coordinated Sampling Campaigns

We undertake regular, coordinated sampling campaigns, both above- and belowground, to determine treatment impacts on plant, microbial and invertebrate communities, and associated changes in ecosystem properties and processes. Details of these sampling campaigns are outlined below, with selected data presented in this methods paper; further data characterizing above- and belowground responses will be presented in subsequent publications.

### Vegetation Monitoring

Non-destructive vegetation cover measurements are conducted approximately every 4 months by placing a 1 m^2^ quadrat with 25 sub-divisions in the center of each plot and recording species level presence/absence data in each sub-division. Since October 2013, twice-yearly harvests (April and October) of all aboveground plant material have been undertaken. For this, vegetation is cut to ground level within the central 1 m^2^ of each plot and, in a randomly selected subsample (20–40% of the harvested material), live (green) material is sorted to species level and separated from dead biomass. All plant material is oven-dried at 80°C for 48 h, and weighed to provide a measure of growing season (October–April) and cool season (April–October) productivity for all plots.

### Invertebrate Monitoring

Immediately prior to the harvests in October 2013, April 2014, and October 2014, aboveground invertebrates were sampled from each of the plots using a ‘G-Vac’ suction sampler (SH 86C, Stihl AG & Co. KG, Germany). The device was passed over the plots in a zigzag pattern for 20 s, with all dislodged material and invertebrates captured in a fitted organza bag. In addition, quarterly from October 2013 until April 2015, yellow sticky card traps (Bugs for Bugs, Mundubbera, QLD, Australia) were suspended from the center of each shelter roof (or at the same height for unsheltered controls) for a period of 1 week to capture flying invertebrates. Invertebrates from both suction samples and sticky traps were identified to at least Order level (except for two groups taken to Subclass only – Acari and Collembola).

To quantify belowground invertebrate responses to altered rainfall regimes, two composite soil samples, each composed of two soil cores (3 cm diameter, 10 cm depth) are collected at the beginning (October) and end (April) of each growing season for extraction of soil nematodes and microarthropods. We focus on these two groups as they are the two most abundant soil invertebrate groups. Nematodes and microarthropods are extracted using standard techniques (Baermann, 1917; Tullgren, 1918). Nematodes are classified to trophic level based on morphology under an inverted microscope, and counts converted to individuals per kg dry soil. Microarthropods are initially sorted into springtails, oribatid, mesostigmatid and other mites (for more detail see Nielsen et al., 2016). More detailed analyses will be undertaken on archived samples over the course of the experiment. Further assessments of soil invertebrate groups that require more destructive sampling campaigns will be undertaken at a later stage in the experiment to avoid substantial disturbance.

### Plant, Soil, and Microbial Analyses

Leaf material was sampled from three grass species (*C. dactylon, E. curvula*, and *M. stipoides*) in November 2014 and analyzed for silicon concentrations. Ground plant material was pressed at 11 tons into 5 mm thick cylindrical pellets with a manual hydraulic press using a 13 mm die (Specac, Orpington, UK). Si concentration (% dry mass) was determined using a commercial P-XRF analyser (Niton XL3t900 GOLDD analyser: Thermo Scientific Winchester, UK) held in a test stand (SmartStand, Thermo Scientific, Winchester, UK; Reidinger et al., 2012).

Since April 2014, we have carried out regular sampling campaigns to investigate treatment effects on bulk soil properties (e.g., chemistry, nutrient availability) and processes (e.g., enzyme activities). Soil samples comprise 8–10 cores (0–10 cm deep, 1 cm wide) per plot. Analyses for soil chemistry, microbial and enzyme activity are conducted using fresh soil samples; molecular analyses (qPCR and MiSeq Illumina high-throughput sequencing) are carried out on DNA extracted from frozen samples, using the PowerSoil kit^®^ (MoBio). Results of soil and microbial analyses will be presented in a subsequent paper.

## Statistical Analysis

All analyses were carried out using linear models in R (Version 3.2.4, R Core Team, 2016). Shelter effects on PAR and air temperature were evaluated for month-long periods in summer (November 2014) and winter (August 2014), to compare differences between AMB (sheltered) control plots and outside (unsheltered) control plots. Data from all 48 sheltered plots were used to evaluate rainfall treatment effects on plant biomass. Data were first inspected for homogeneity of variances and normality of errors and, where necessary, log, box-cox or arc-sine transformation was carried out prior to analyses (Crawley, 2012). Treatment effects were evaluated by first fitting the full model (rainfall treatment, herbivore addition and their interactions) and then model simplification was undertaken by removing non-significant terms. When neither the interaction between rainfall treatment and herbivore addition, nor herbivore addition on its own were significant (i.e., *P* > 0.10), herbivore-added plots were retained in the analysis to assess rainfall treatment effects. When overall treatment effects were significant, Tukey’s HSD *post hoc* tests were used to determine significance between treatment levels; results were considered significant if *P* < 0.05.

Soil moisture data (November 27, 2013 to November 25, 2014) obtained from automatic sensors were averaged per week and the effects of rainfall treatment were evaluated with a repeated-measures linear mixed-effects model [lme in the nlme package (Pinheiro et al., 2016)] with plot nested within treatment as a random effect. In order to test for the effect of root herbivore addition, generalized linear mixed models were constructed with the lmer() function in the lme4 package (Bates et al., 2015), and Chi square (χ^2^) values between models with and without the herbivore interaction were compared (Faraway, 2006). *Post hoc* comparisons were performed with glht() in the multcomp package with a ‘Benjamini–Hochberg’ correction (Hothorn et al., 2008).

The effect of watering treatment on aboveground invertebrate abundance was assessed using linear models on square-root transformed abundance data. Watering treatment was included in the model as an independent variable along with scaled plot biomass, given the documented effect of underlying plant structure on sampling efficiency (Facey and Torode, 2016). Effects of root herbivore addition on the presence/abundance of scarabs in the soil were analyzed with a zero-inflated-poisson model in the *pcsl* package, and model significance evaluated using a likelihood ratio (lr) test (Jackman, 2015).

## Results and Discussion

### Shelter Effects on Microclimate

Differences in air temperature between unsheltered and sheltered plots varied diurnally and between seasons (**Table 2**). On average (24 h mean), sheltered plots were 0.04°C warmer than unsheltered ones, representing non-significant daytime cooling and nighttime warming associated with shelter roofs; this phenomenon is well known from previous studies using permanently installed shelter infrastructure (Fay et al., 2000; Beier et al., 2004; Vogel et al., 2013). Whilst temperature was only minimally affected by the presence of shelter roofs, effects on PAR were more substantial. On average, PAR was significantly lower under shelters than in OPs (-15.9%; *F*_1,2_ = 145.3, *P* < 0.01). Interception losses averaged 17.4% during summer months (*F*_1,2_ = 139.5, *P* < 0.01) and 13.1% in winter (*F*_1,2_ = 198.9, *P* < 0.01). This is directly comparable to light interception values reported for similar studies in Germany (15%, Vogel et al., 2013) and the USA (21%, Fay et al., 2000) where, like ours, shelter roofs cover the entire plot area. Lower levels of PAR interception have been associated with shelter infrastructure where roofs cover a smaller proportion of the plot area. For example, Gherardi and Sala (2013) report reductions of just 3 and 6% for shelters covering 50 and 80% of the plots, respectively, while Yahdjian and Sala (2002) found a 10% reduction in PAR associated with roofs covering 80% of the plot area.

**Table 2:** Shelter effects on canopy air temperature and photosynthetically active radiation (PAR).

		Air temperature (°C)			PAR (mean daily mol m^-2^)		
		Outside	Shelter	Diff (°C)	Outside	Shelter	Diff (%)
Overall	24 h	15.73	15.77	+0.04	-	-	-
	Daylight hours	19.43	19.24	-0.19	34.98	29.43	-15.9%
	Night time	12.04	12.30	+0.26	-	-	-
Summer (November)	24 h	20.46	20.48	+0.02	-	-	-
	Daylight hours	23.70	23.55	-0.15	41.54	34.30	-17.4%
	Night time	17.23	17.42	+0.19	-	-	-
Winter (August)	24 h	11.15	11.21	+0.06	-	-	-
	Daylight hours	15.30	15.08	-0.22	27.41	23.80	-13.1%
	Night time	7.01	7.35	+0.34	-	-	-

Light interception is an unavoidable artifact of field experiments involving fixed roofs. Unless within-shelter PAR is above light-saturation levels for much of the growing season (e.g., Fay et al., 2000), shelter-induced reductions in PAR are likely to have implications for photosynthesis and, depending on other resource constraints, potentially also productivity. Whilst we only measured PAR, it is worth noting that other shelter-associated changes in spectral characteristics can also influence other photosensitive ecosystem processes. For example, Vogel et al. (2013) attributed differences in litter decomposition rates and plant metabolic profiles to contrasting levels of UV radiation associated with shelter roofs in a recent rainfall manipulation experiment, advocating for the need to include roofed controls in shelter-based studies.

Outside plots had slightly higher soil water content (SWC) compared to sheltered AMB plots (**Table 3**), although differences were not statistically significant (χ^2^ = 0.254, df = 1, *p* = 0.614). Given the link between canopy transpiration rates and SWC (Patrick et al., 2007), these differences may be due to slightly higher transpirational water loss associated with greater vegetation biomass in AMB compared to OP (see below). A second possible explanation for these differences could be the method for water delivery to plots. The relatively small droplet size of water applied via sprinklers increases the chance of both spray drift and higher levels of canopy interception (and subsequent evaporation; Moss and Green, 1983), both of which could result in lower SWC for a given water application, compared to natural rainfall.

**Table 3:** Mean seasonal and annual volumetric soil water content (SWC, %) and seasonal rainfall (mm), 2013–2014.

Treatment	Winter	Spring	Summer	Autumn	Annual
Ambient (sheltered)	14.0 (0.49)^a^	10.3 (0.45)^a^	10.0 (0.32)^a^	13.0 (0.34)^a^	11.8 (0.22)^a^
Reduced amount	12.2 (0.46)^b^	9.2 (0.48)^a^	8.6 (0.20)^a^	10.0 (0.23)^b^	9.9 (0.19)^b^
Increased amount	13.0 (0.51)^a^	10.2 (0.55)^a^	9.8 (0.43)^a^	13.0 (0.43)^a^	11.4 (0.25)^ab^
Reduced frequency	11.1 (0.43)^b^	7.7 (0.40)^a^	8.6 (0.45)^a^	12.5 (0.40)^a^	10.0 (0.23)^ab^
Summer drought	13.8 (0.50)^a^	10.9 (0.51)^a^	8.7 (0.30)^a^	7.3 (0.03)^b^	10.0 (0.22)^ab^
Treatment effects (df = 1,4)	χ^2^ = 23.5, *P* = 0.0001	^χ^2^^ = 7.85, *P* = 0.097	^χ^2^^ = 8.06, *P* = 0.089	^χ^2^^ = 21.4, *P* = 0.0003	^χ^2^^ = 15.3, *P* = 0.009
Outside plots (unsheltered)	14.3 (0.55)	11.4 (0.55)	10.5 (0.36)	15.3 (0.45)	12.8 (0.26)
**Ambient rainfall (mm)**					
06/2013–05/2014	80.4	230.9	124.7	160.6	596.7
30-years mean	137.6	182.4	280.7	205.6	806.3
30-years CoV	77.3%	41.4%	43.4%	60.8%	26.1%

*Values in brackets represent ±1 SE. Rainfall treatment effects on SWC are evaluated for all sheltered plots (i.e., excluding unsheltered control plots). Ambient rainfall means and coefficients of variation (CoV) also summarized by season, for the past 30 years (1982–2012). Different letters indicate significant differences between treatments.*

Soil water content within 25 cm of the edge of RA, RF, and SD plots was typically 0–0.5% higher than in the center of the plot. Immediately after heavy ambient rainfall episodes, differences of up to 2.8% were noted, but overall differences in SWC between the center and outside 25 cm of the plot area were small. The biggest differences were observed in SD plots, following a large rainfall event during the period of total rainfall exclusion, when within-plot SWC was particularly low. At this time, average SWC was 23.9% outside of these shelters, while values within SD plots ranged from 2.5% in the plot center, to 3.3 and 6.3% at distances of 50 and 25 cm from the outer edge of the plots, respectively. In the context of ambient rainfall incursion, we estimate the size of the edge effect to be approximately 25 cm. This is directly comparable with values reported for similar shelters elsewhere (e.g., 20 cm; Gherardi and Sala, 2013), and confirms that the combination of roof interception, impermeable root barrier and a well-drained, sandy soil provide effective hydrological isolation of our experimental plots under all but the wettest/windiest conditions.

### Shelter Effects on Plant Productivity

**FIGURE 2:**
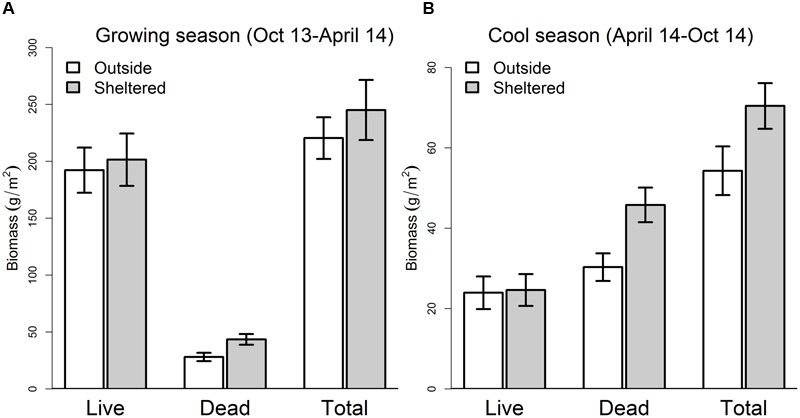
**Harvested plant biomass in sheltered ambient and outside (unsheltered) plots in **(A)** April 2014 (growing season) and **(B)** October 2014 (cool season)**.

The differences in SWC, air temperature and PAR between AMB and OP were associated with modest differences in ANPP. Growing season ANPP was 10.8% higher, and cool season ANPP was 29.7% higher in AMB compared to OP (**Figure 2**), although neither of these differences were statistically significant. The larger shelter effects on cool season productivity were driven by a significantly greater accumulation of dead plant material in AMB plots (+51%; *F*_1,22_ = 7.87, *P* < 0.001). Although shelter impacts on ANPP were not statistically significant, the biological relevance of 10–30% differences in productivity is arguably high and emphasises the need to compare treatment effects to sheltered controls (AMB). The importance of controlling for shelter artifacts has been raised in rainfall manipulation studies elsewhere, with shelter infrastructure associated with altered net primary productivity (NPP), decomposition and carbon fluxes (Fay et al., 2000; Vogel et al., 2013). Based on information on how shelters modify the microclimate in our study, and associated biological responses, all rainfall and herbivore treatment effects are evaluated against sheltered AMB plots, with unsheltered plots used to provide a context for interpreting these effects.

### Treatment Effects and Seasonal Patterns in Soil Water Content

**FIGURE 3:**
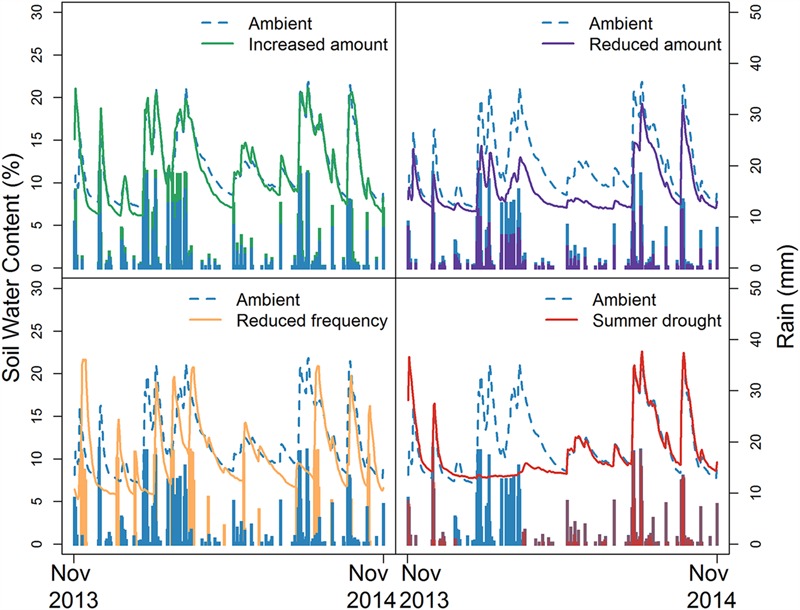
**Temporal trends in soil water content, by treatment, from November 2013 to November 2014**.

Ambient rainfall at the site for the 12-months period from June 2013 to May 2014 was 597 mm, lower than the 30 years mean of 806 mm. During the 1st year of the experiment, summer rainfall was particularly low, with less than half the long-term seasonal average falling in the local area. Temporal trends in SWC are illustrated in **Figure 3**. Treatment differences reflect both the timing of ambient rainfall and that of imposed treatments with, for example, the 3-weekly periodicity of the RF treatment, and the summer-long water withholding in the SD treatments, clearly reflected in soil moisture patterns.

**Table 3** summarizes overall and seasonal treatment effects on SWC for the first 12 months of the experiment. The biggest differences were seen during the summer (December–February), corresponding to the period of maximum plant growth and the timing of the SD treatment. There was a significant overall effect of rainfall treatment on SWC but no effects of herbivore addition, nor an interaction between the two treatments. *Post hoc* analyses revealed that moisture levels were higher in AMB compared to RA plots; RF experienced greater variation in soil moisture, with periods where SWC was higher and others where it was lower than the other treatments, during the 21-day watering cycle. The lowest seasonal mean SWCs were associated with different treatments in different seasons; in winter and spring RF plots had the driest soils, while in autumn SD had the lowest SWC.

Annual mean SWC was consistent between all reduced rainfall treatments (RA, RF, and SD) and clearly demonstrates that contrasting rainfall regimes can result in similar long-term mean SWC, despite highly contrasting patterns both within- and between- seasons. Increasing rainfall variability (i.e., longer inter-pulse intervals) has been associated with increased (or decreased) mean SWC, depending on background climatic conditions and soil type (Zeppel et al., 2014). Under mesic conditions, reducing the frequency of rainfall events (with no change in total rainfall amount) has been found to lower mean SWC (Harper et al., 2005; Fay et al., 2011), but in arid systems similar reductions in frequency can actually increase mean SWC, particularly in deeper soil horizons (Heisler-White et al., 2008, 2009). With a long-term mean rainfall of 806 mm for the local area, SWC in the RF treatment in our study parallels that at other mesic sites and highlights the importance of changes in the pattern, as well as the amount of rainfall for ecosystem hydrology under climate change.

Unlike field-based rainfall manipulations elsewhere (Fay et al., 2000; Gherardi and Sala, 2013), differences in seasonal means (**Table 3**) and temporal patterns (**Figure 3**) in SWC between AMB and IA treatments at our site were minimal. This likely reflects greater transpirational water loss associated with higher plant biomass in IA, and the high drainage capacity and relatively low soil water-holding capacity (Atwell et al., 1999) of our sandy soils, compared to other studies (e.g., silty clay loam; Fay et al., 2000). It also emphasizes that impacts of future shifts in rainfall regime will be contingent not only on the nature of the change, but will also depend on the climate context and soil conditions at a given site.

### Early Vegetation Responses to Rainfall and Root Herbivore Treatments

**FIGURE 4:**
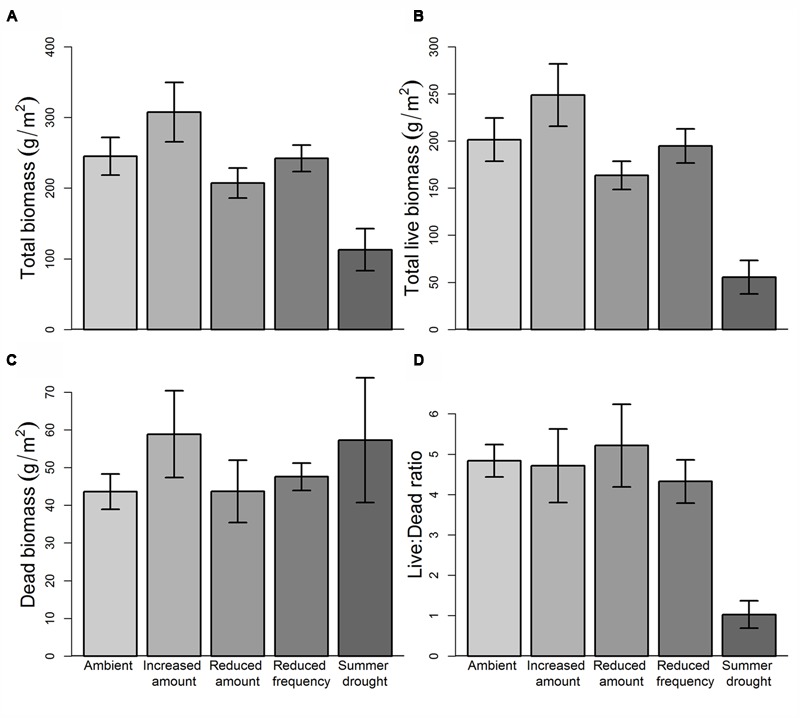
**Rainfall treatment effects on growing season biomass (April 2014 harvest). (A)** Aboveground NPP (October–April), **(B)** live biomass, **(C)** dead biomass, **(D)** live:dead biomass ratio. Values are means ±1SE.

Total ANPP in the first growing season (October 13–April 14) was significantly affected by rainfall treatment (*F*_4,43_ = 7.70, *P* = 9.03e^-05^), but there was no effect of herbivore addition, nor interactions between rainfall and herbivore treatments at this time. *Post hoc* comparisons reveal that rainfall effects on ANPP were driven primarily by a significant reduction (-62.3%, *P* = 0.0004) in biomass in SD plots (168.4 ± 46.2 g m^-2^) compared to AMB (446.6 ± 49.4 g m^-2^, **Figure 4A**). ANPP in IA and RA treatments were not significantly different from AMB, but there was a clear gradient in productivity, increasing from 370.9 (±35.8) g m^-2^ in RA to 556.3 (±74.7) g m^-2^ in IA. This represents a positive linear relationship between ANPP and water inputs for these treatments, despite the absence of a clear relationship with mean SWC. ANPP in RF plots was similar to AMB, despite a somewhat higher mean SWC in RF plots.

Treatment effects on live (green) harvested biomass in April were very similar to those for total aboveground productivity, with a significant overall effect of rainfall (*F*_4,43_ = 6.20, *P* = 0.0005) but not herbivore addition, nor interactions between the two treatments (**Figure 4B**). The amount of dead plant material harvested at the end of the growing season was fairly consistent across plots, with no significant treatment effects (**Figure 4C**). However, the ratio of live to dead material differed significantly (*F*_4,43_ = 3.76, *P* = 0.0104) between contrasting rainfall regimes, with dead material representing 17.8% of total aboveground biomass in AMB plots, but 58.8% in SD plots (**Figure 4D**).

Taken together, these early data indicate that the total amount of growing season rainfall is a more important determinant of vegetation productivity at our site than the frequency of those inputs. Close relationships between rainfall amount and plant growth are well established (Sala et al., 1988; Hsu et al., 2012; Southon et al., 2012). However, the lack of biomass response to altered rainfall frequency contrasts with recent studies that report negative impacts on species productivity, cover and nutritional quality (Walter et al., 2012; Jones et al., 2016), as well as greater impacts on ecosystem processes, than reducing total rainfall amount in both mesic (Heisler-White et al., 2009; Fay et al., 2011) and (semi-) arid grasslands (Heisler-White et al., 2008; de Dios Miranda et al., 2009). In our study, plant community resistance to altered rainfall frequency may reflect the high variability in rainfall; coefficients of variation in seasonal rainfall are naturally high (particularly during spring) at our site compared to other sites (e.g., Walter et al., 2012) and it is likely that the vegetation has adapted to historically high levels of rainfall variability. The potential for changes in plant community composition to buffer changes in ecosystem functioning under more variable rainfall conditions (Fry et al., 2013, 2014b; Gherardi and Sala, 2015) may also explain the lack of biomass response to RF treatment in our study, and will be a subject for future investigation.

**FIGURE 5:**
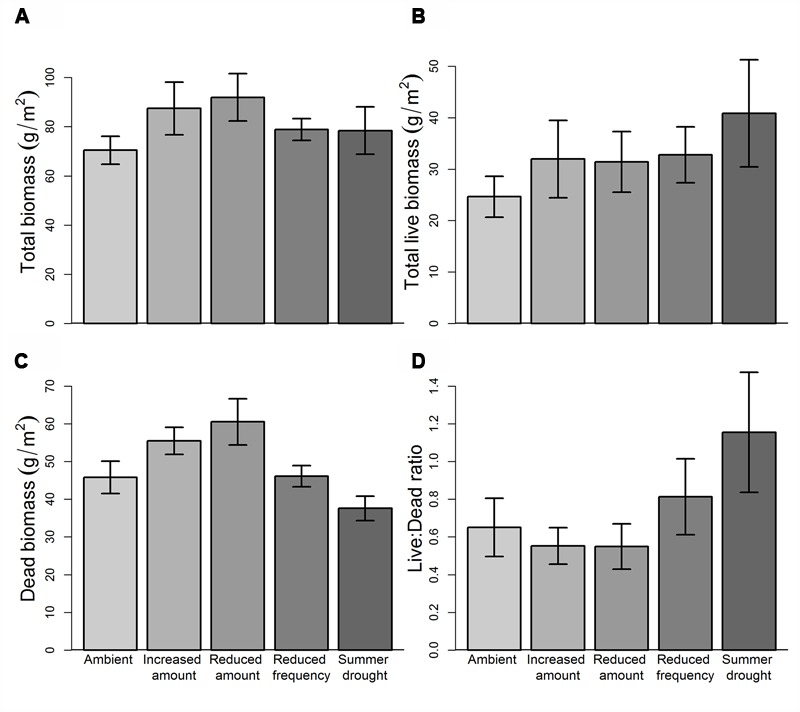
**Rainfall treatment effects on cool season biomass (October 2014 harvest). (A)** Aboveground NPP (April–October), **(B)** live biomass, **(C)** dead biomass, **(D)** live:dead biomass ratio. Values are means ±1SE.

Cool-season (April–October) ANPP and live biomass were not affected by either rainfall or herbivore addition treatments, or their interactions (**Figure 5**). Treatment effects on dead biomass were only significant for rainfall (*F*_4,43_ = 3.329, *P* = 0.018), with more dead plant material in RF (+32.1%, *P* = 0.017) than AMB at this time. Although not statistically significant, there was nearly twice as much live plant material in SD plots in the October harvest as in AMB (*P* = 0.096), demonstrating very rapid vegetation recovery once the summer-long drought was released. This, together with levels of cool-season productivity in all water-manipulated treatments that were higher than AMB plots, implies a high degree of climate resilience at our site. The ability for water-stressed ecosystems to recover is likely associated with rapid recovery of formerly dominant species, or compensatory growth by other (previously sub-ordinate or newly recruited) species within these plots. Previous rainfall manipulation studies have shown contrasting rates of recovery, with evidence of both rapid return to pre-drought levels of ANPP (Hoover et al., 2014) and legacy effects persisting for many years (Haddad et al., 2002; Sala et al., 2012). Shifts in plant community composition represent a key mechanism by which physiologically driven decline in NPP under drought can be offset (Hoover et al., 2014; Gherardi and Sala, 2015). Compositional change will, therefore, be closely monitored at our site over the next 3–5 years to establish the relationship between diversity, community-weighted functional traits and both resistance and resilience to rainfall change.

The absence of effects of root herbivore addition on plant productivity responses is not surprising, given the timing of additions (December 2013 and February/March 2014) in relation to the first growing season (October 2013–April 2014). Furthermore, given scarab preferences for grazing on more nutritious grass species (e.g., C_3_ species; Johnson et al., 2014), shifts in community composition may be more likely than impacts on plot-level productivity. Other studies (e.g., Schallhart et al., 2012) report root herbivore-associated plant community change, and this may become more apparent in our study over time.

### Invertebrate Responses

Root herbivore treated plots contained significantly higher abundances of root-feeding insects (mostly scarabs) than those that were not inoculated [23.3 m^-2^ ± 9.9_(0-20__cm depth)_ and 5.6 m^-2^ ± 2.7_(0-20__cm depth)_, respectively] (Log-likelihood_5_ = -39.6, *P* = 0.0052).

Preliminary results from the aboveground invertebrate sampling campaigns found that invertebrate abundance was not significantly influenced by the imposed rainfall regime (**Table 4**). However, this lack of response in the invertebrate community regimes is not surprising after only four months of treatments and may change as more data become available from subsequent sampling campaigns. In particular, we may expect invertebrate abundance aboveground to be negatively affected by the reductions in plant material occurring when the SD treatment is imposed (December–March).

**Table 4:** Mean total aboveground invertebrate abundances (individuals) from the first sampling campaign (October 2013).

	Mean total aboveground invertebrate abundance	
Rainfall treatment	Sticky traps	Vacuum samples
Ambient	216.8 (20.2)	133.8 (36.8)
Increased amount	209.3 (18.9)	225.5 (61.9)
Reduced amount	233.4 (19.0)	165.8 (43.4)
Reduced frequency	227.8 (15.2)	396.5 (143.0)
Summer drought	237.5 (27.0)	230.0 (59.7)
Rainfall	*F*(4,42) = 0.391	*F*(4,42) = 0.962
	*P* = 0.814	*P* = 0.438

*Values in brackets represent ±1 SE.*

No effects of altered precipitation were observed in terms of the abundances of nematodes, nematode trophic group or microarthropods after more than 1.5 years’ climate manipulation (i.e., April 2015; Nielsen et al., 2016). However, there were subtle, significant changes in nematode feeding guild composition and diversity in SD plots, suggesting that nematodes are sensitive to extreme events in this grassland (Nielsen et al., 2016). Similar results have been observed in other studies (e.g., Cesarz et al., 2015). These responses will be investigated in depth later in the experiment, to determine if belowground invertebrate responses are amplified or ameliorated over time.

**FIGURE 6:**
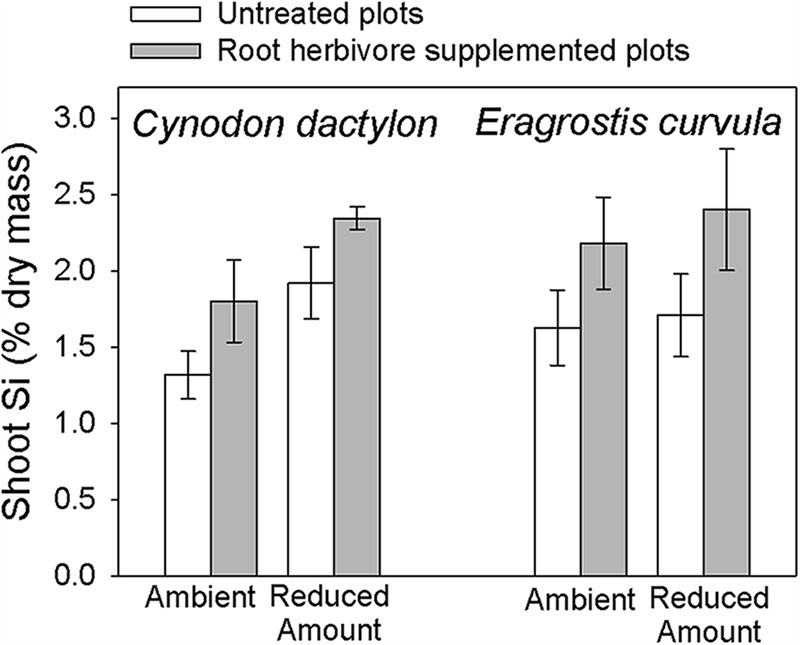
**Effects of root herbivore addition treatment on foliar silicon concentrations in Cy*nodon dactylon* and *Eragrostis curvula***.

A number of plant chemical characteristics have been measured, but here we focus on silicon (Si) concentrations because grasses typically accumulate high levels of Si and this has been shown to increase their resistance to both abiotic (e.g., drought) and biotic (e.g., herbivory) stress (Epstein, 1999; Cooke and Leishman, 2011). In particular, Si has been demonstrated to be an inducible defense against aboveground herbivores (Massey et al., 2007). We found similar patterns of induction in two of our three sampled grasses, *C. dactylon* and *E. curvula*, in response to belowground herbivore addition (**Figure 6**). To our knowledge, this is the first example of belowground herbivores inducing this defense in grasses. Future work will report whether this effect persists and whether rainfall treatments moderate the induction of this important plant defense.

## Conclusion

This paper introduces a new experimental platform that, uniquely, combines multi-level rainfall manipulation with contrasting levels of root herbivory. Early data clearly identify the importance of shelter controls in rainfall manipulation experiments of this type, in order to assess potential shelter artifacts that may otherwise obscure treatment effects. This SE Australian grassland exhibited relatively high resistance of NPP to changes in the size and frequency of rainfall inputs, except under extreme SD. The rapid recovery of NPP in SD plots after ambient rainfall inputs were resumed indicates that low ecosystem resistance to climate extremes is not necessarily associated with low functional resilience. This may reflect adaptation of the plant community to the naturally high variability in rainfall that can occur both between- and within- years in Australia, with annual inputs at our site varying by as much as 66% below and 114% above the long-term mean. The absence of a productivity response to herbivore addition may be a consequence of the timing of this treatment in relation to the first growing season, compensatory growth by affected plant species and/or changes in plant community composition. This research platform will allow ongoing monitoring of ecological responses to novel combinations of abiotic and biotic stresses, and identification of mechanisms underlying observed above- and belowground responses.

One of the biggest challenges in ecosystem ecology today is to improve our understanding of the mechanisms by which plant physiological and morphological responses to climate change affect interactions within- and between- trophic levels, and ecological feedbacks (Van der Putten et al., 2010). The DRI-Grass experimental platform provides the opportunity to gain important new insight into how ecological interactions are affected by changing rainfall regimes and, specifically, how belowground herbivory modifies grassland resistance and resilience to climate extremes.

## Author Contributions

SP, SJ, UN, and DT designed and led set up of the experimental facility. SP contributed to data collection and analysis, and led overall data interpretation and writing. UN led nematode/microarthropod data collection, analysis and interpretation and assisted with writing. SJ led plant Si data analysis and interpretation and assisted with writing. DT contributed to data interpretation and writing. KB implemented root herbivore treatments, led collection of vegetation and scarab data, contributed substantially to data analysis and also writing. RO-H and EG-F contributed to field data collection and assisted with writing. SF led aboveground invertebrate sampling and analysis, and contributed to writing. SH ran foliar Si analyses, and contributed to data interpretation and writing.

## Conflict of Interest Statement

The authors declare that the research was conducted in the absence of any commercial or financial relationships that could be construed as a potential conflict of interest.

## Footnotes

1 http://www.bom.gov.au
