# Supplementary material for: DRI-Grass: A New Experimental Platform for Addressing Grassland Ecosystem Responses to Future Precipitation Scenarios in South-East Australia
Source: Front Plant Sci. 2016 Sep 20;7:1373. doi: 10.3389/fpls.2016.01373 (PMC5028386; doi:10.3389/fpls.2016.01373)
Supplement: Supplementary file 1 [file Table_1.DOCX]

Table S1. Species recorded at the experimental site to date. * indicates those species that represent at least 5% of harvest biomass or >5% cover during vegetation surveys.

| *Ambrosia artemisiifolia* | |  | *Lotus angustissimus* | |  |
| --- | --- | --- | --- | --- | --- |
| *Anagallis arvensis* | |  | *Lotus corniculatus* | |  |
| *Aster subulatus* | |  | *Medicago sativa* | |  |
| *Avena fatua* |  |  | **Microlaena stipoides* | |  |
| **Axonopus fissifolius* | |  | *Modiola caroliniana* | |  |
| *Bidens pilosa* | |  | *Oenothera stricta* | |  |
| *Bothriochloa macra* |  |  | *Ornithopus compressus* | |  |
| *Briza subaristata* | |  | *Oxalis corniculata* | |  |
| *Bromus catharticus* | |  | *Oxalis perennans* | |  |
| *Carex inversa* | |  | *Paspalidium distans* | |  |
| *Centaurium tenuiflorum* | |  | *Paronychia sp.* | |  |
| *Cichorium intybus* | |  | **Paspalum dilatatum* | |  |
| *Cirsium vulgare* | |  | **Paspalum notatum* | |  |
| *Commelina cyanae* | |  | *Petrorhagia velutina* | |  |
| *Conyza sumatrensis* | |  | **Plantago lanceolata* | |  |
| **Cymbopogon refractus* | |  | *Portulaca oleracea* | |  |
| **Cynodon dactylon* | |  | *Romulea rosea* | |  |
| *Cyperus sesquiflorus* | |  | **Senecio madagascariensis* | |  |
| *Dichelachne spp.* | |  | **Setaria parviflora* | |  |
| *Digitaria didactyla* | |  | *Sida rhombifolia* | |  |
| *Digitaria sanguinalis* | |  | *Solanum nigrum* | |  |
| **Eragrostis curvula* | |  | *Sonchus oleraceus* | |  |
| *Gamochaeta pensylvanica* | |  | *Sporobolus africanus* | |  |
| *Hydrocotyle peduncularis* | |  | *Swainsona adenophylla* | |  |
| *Hypericum gramineum* | |  | *Trifolium repens* | |  |
| **Hypochaeris radicata* | |  | *Verbana spp.* | |  |
| *Hypochaeris sumatrensis* | |  | *Vicia sativa* |  |  |
| *Juncus cognatus* | |  | *Viola betonicifolia* |  |  |
| *Leontodon taraxacoides* | |  | *Vulpia spp.* | |  |
| *Lolium perenne* | |  | *Zornia dyctiocarpa* |  |  |
|  | |  |  | |  |
